# Single-port robot-assisted cervical esophagectomy (SP RACE): Combining precision mediastinal lymphadenectomy and complete extrapulmonary dissection

**DOI:** 10.1016/j.xjtc.2025.07.010

**Published:** 2025-09-18

**Authors:** Franziska Renger, Luca Bellaio, Edin Hadzijusufovic, Vladimir J. Lozanovski, Hauke Lang, Peter P. Grimminger

**Affiliations:** Division of Upper GI Surgery, Department of General, Visceral and Transplantation Surgery, University Medical Centre of the Johannes Gutenberg-University Mainz, Mainz, Germany

**Figure:**
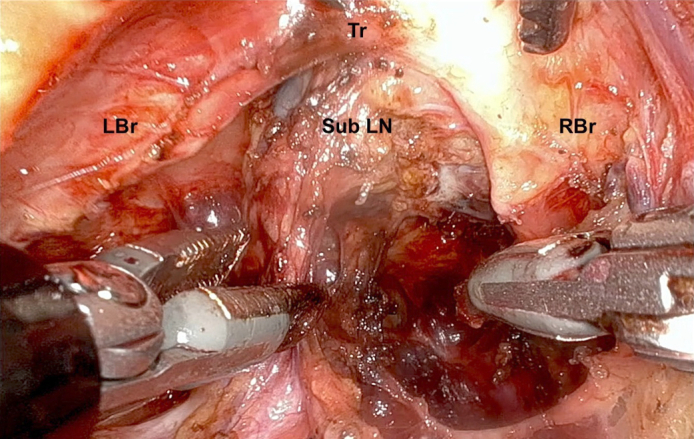
Mediastinal lymphadenectomy - subcarinal lymph nodes.

**Central Message**

SP RACE enables precise robot-assisted mediastinal lymphadenectomy and esophagectomy by pure extrapulmonary dissection, offering extended surgical treatment options for patients with pulmonary disease.

The da Vinci single-port robot-assisted cervical esophagectomy (dV SP RACE) procedure is a new asset within the minimally invasive esophagectomy portfolio in clinic using the da Vinci SP system (Intuitive 2024). Several mediastinoscopic techniques have preceded the approach presented here.^1^, ^2^, ^3^, ^4^, ^5^

In patients considered unfit for single-lung ventilation, transhiatal dissection is a viable option. However, whether it is oncologically adequate concerning mediastinal lymphadenectomy remains unclear. SP RACE combines a cervical, mediastinal, and abdominal procedure,^1^^,^^6^^,^^7^ avoiding the transthoracic route but facilitating lymphadenectomy (Video 1). Neither single-lung ventilation nor thoracotomy are needed. An approach with two teams could further shorten operation time. We believe 3-dimensional, magnified vision and reduced robotic arm collision enable superior triangulation and precise dissection, especially in narrow spaces such as the posterior mediastinum. Currently only the da Vinci SP system allows surgeons to reach the lower mediastinum from the neck (Video 2).

The technical complexity of controlling the SP robot and an unusual angle of an already-complex anatomical region should not be underestimated.^4^^,^^8^ As the result of bipulmonary ventilation, a collapsing operation field needs to be managed. With sealing devices missing, control of bleeding is more challenging, and coagulation smoke can impair vision. Inherent to a single-port set-up, changing of instruments takes longer and entails repeat set-up of the operation field.

### Indication

SP RACE is indicated for patients who require esophagectomy with mediastinal lymphadenectomy for esophageal cancer with or without neoadjuvant treatment. It is particularly useful for mid- and upper mediastinal tumors and patients considered unfit for transthoracic surgery because of compromised lung function or previous thoracic surgery: SP RACE could extend surgical options for these patients.

### Set-Up and Access

The patient is in the supine position throughout the entire procedure. The patient cart is positioned on the right side of the patient (Video 3). To start the cervical phase, a 4-cm incision is made alongside the lower third of the left sternocleidomastoid muscle. Further preparation gives access to the carotid sheath. The left vagus nerve is isolated and continuous neuromonitoring (Inomed) is established. Preparation continues medially to the vascular bundle to reach the left esophageal wall. Cervical lymphadenectomy can be performed. The left recurrent laryngeal nerve is identified. The access port is inserted, a capnomediastinum at 8 mm Hg established, and the SP da Vinci system is docked. A suction device via the access port allows suctioning of blood and smoke.

### Left-Sided Dissection

Robotic dissection starts on the left side of the esophageal wall. The left recurrent laryngeal nerve is visualized (Video 4). The lateral dissection plane is developed between the visceral fascia of the esophagus and the left mediastinal pleura until the aortic arch is reached. Lymph nodes alongside the left recurrent laryngeal nerve and in the aortopulmonary window are cleared, optionally. The left main bronchus is delineated, and corresponding lymph nodes resected. At the level of the aortic arch, the esophagus is released from the aortoesophageal ligament.^8^

### Dorsal Dissection

In the upper mediastinum, dorsal dissection is guided by the esophagus anteriorly and the prevertebral fascia posteriorly (Video 5). The thoracic duct crosses to the left.^8^ Indocyanine green visualization facilitates the identification of its trajectory, ensuring preservation and avoiding chyle leaks (Video 6).

### Ventral Dissection

The ventral dissection plane is created between esophagus and trachea (Video 7). Separating the esophageal muscular tube and pushing the fascial envelope toward the trachea also protects the left recurrent laryngeal nerve.^8^ For exposure, the trachea needs to be lifted carefully to avoid injure to the membranous part. Dissection continues toward the carina.

### Right-Sided Dissection

The right upper mediastinal dissection plane is developed between the visceral fascia of the esophagus and the mediastinal pleura (Video 8). The azygos vein, the right bronchial artery, and the right vagus nerve with its cardiac branches serve as landmarks. Right main bronchus lymph nodes are cleared.

### Subcarinal Lymphadenectomy

Lymph nodes alongside the left and right main bronchus are cleared, as well as subcarinal lymph nodes (Video 9).

### Lower Mediastinal Dissection

Below the subcarinal level, the esophagus and paraoesophageal lymph nodes are dissected circumferentially: ventrally from the pulmonary veins and pericardium, laterally from the mediastinal pleurae and dorsally from the descending aorta until the hiatus is reached (Video 10). Direct tributaries to the esophagus are ligated with clips. The thoracic duct is visualized with indocyanine green and preserved (Video 6).

### Connecting the Transcervical Mediastinal and Abdominal Phase and Anastomosis

Simultaneous to the mediastinal dissection, laparoscopic gastric mobilization, abdominal lymphadenectomy, and formation of the gastric tube are performed (Video 2 and Video 11). The robot is undocked, and the gastric conduit maneuvered up. The proximal esophagus is released, sparing the right recurrent laryngeal nerve. An esophagogastric anastomosis is performed at the cervical level. Closure of the platysma and skin mark the end of the procedure.^7^

## Conflict of Interest Statement

P. P. Grimminger serves as a proctor for Intuitive Surgical. All other authors reported no conflicts of interest.

The *Journal* policy requires editors and reviewers to disclose conflicts of interest and to decline handling or reviewing manuscripts for which they may have a conflict of interest. The editors and reviewers of this article have no conflicts of interest.

## Supplementary Data

Video 1 Feasibility of SP RACE mediastinal lymphadenectomy. Video available at: https://www.jtcvs.org/article/S2666-2507(25)00323-2/fulltext.

Video 2 Connecting the SP robotic mediastinal and laparoscopic abdominal phase. Video available at: https://www.jtcvs.org/article/S2666-2507(25)00323-2/fulltext.

Video 3 SP RACE set-up, cervical access, and port placement. Video available at: https://www.jtcvs.org/article/S2666-2507(25)00323-2/fulltext.

Video 4 SP robotic upper mediastinal left-sided esophageal dissection and left-sided recurrent laryngeal nerve lymphadenectomy. Video available at: https://www.jtcvs.org/article/S2666-2507(25)00323-2/fulltext.

Video 5 SP robotic upper mediastinal dorsal esophageal dissection. Video available at: https://www.jtcvs.org/article/S2666-2507(25)00323-2/fulltext.

Video 6 Visualization of the thoracic duct with ICG. Video available at: https://www.jtcvs.org/article/S2666-2507(25)00323-2/fulltext.

Video 7 SP robotic upper mediastinal ventral esophageal dissection. Video available at: https://www.jtcvs.org/article/S2666-2507(25)00323-2/fulltext.

Video 8 SP robotic upper mediastinal right-sided esophageal dissection. Video available at: https://www.jtcvs.org/article/S2666-2507(25)00323-2/fulltext.

Video 9 SP robotic subcarinal lymphadenectomy. Video available at: https://www.jtcvs.org/article/S2666-2507(25)00323-2/fulltext.

Video 10 SP robotic low mediastinal dissection of the esophagus. Video available at: https://www.jtcvs.org/article/S2666-2507(25)00323-2/fulltext.

Video 11 Laparoscopic gastric conduit pull-up.Video available at: https://www.jtcvs.org/article/S2666-2507(25)00323-2/fulltext.
