# Supplementary figures and images for: Single-port robot-assisted cervical esophagectomy (SP RACE): Combining precision mediastinal lymphadenectomy and complete extrapulmonary dissection
Source: JTCVS Tech. 2025 Sep 18;34:249–52. doi: 10.1016/j.xjtc.2025.07.010 (PMC12683056; doi:10.1016/j.xjtc.2025.07.010)

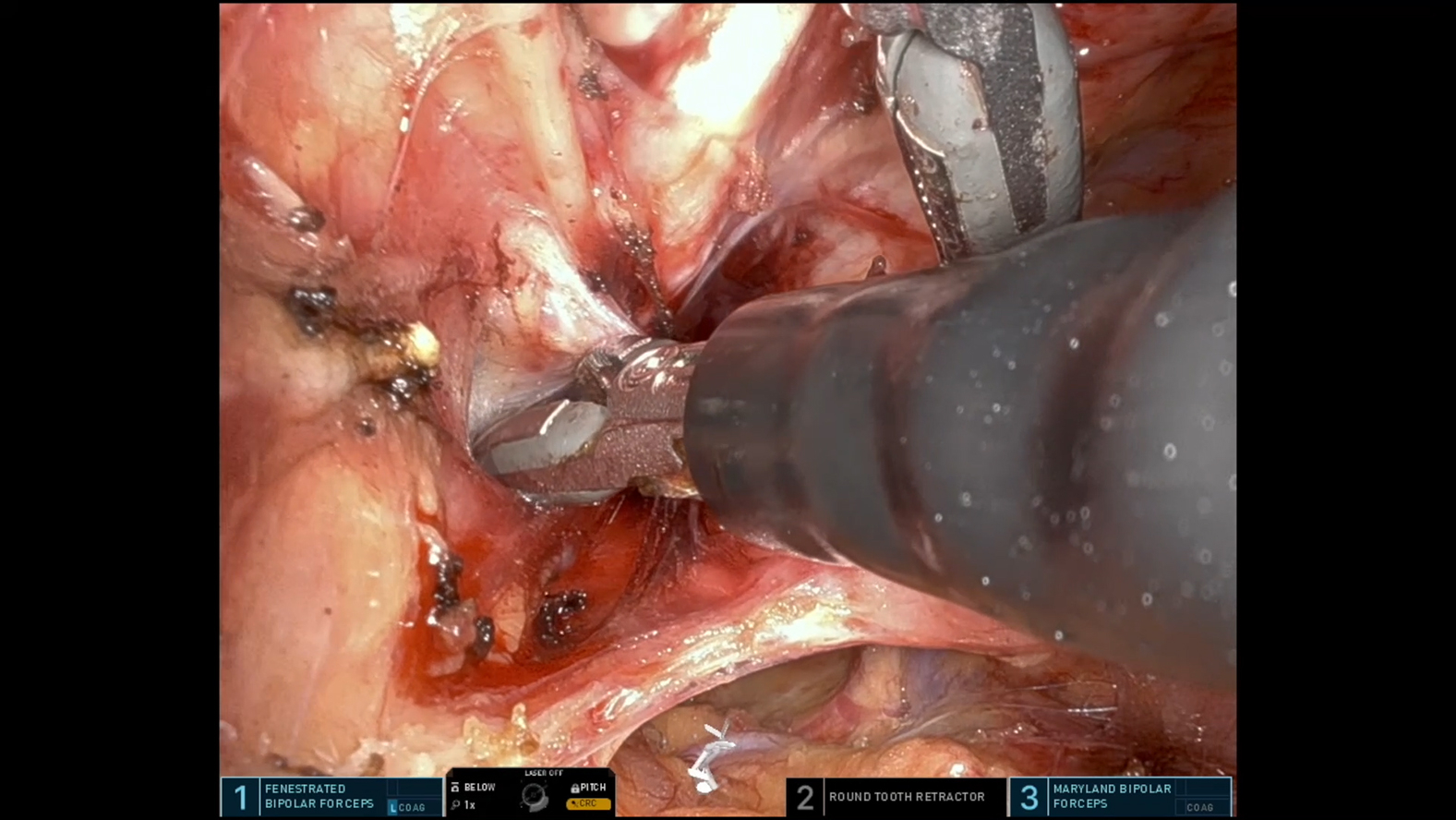

Supplement: Video 1 — Feasibility of SP RACE mediastinal lymphadenectomy. Video available at: https://www.jtcvs.org/article/S2666-2507(25)00323-2/fulltext. [file fx2.jpg]

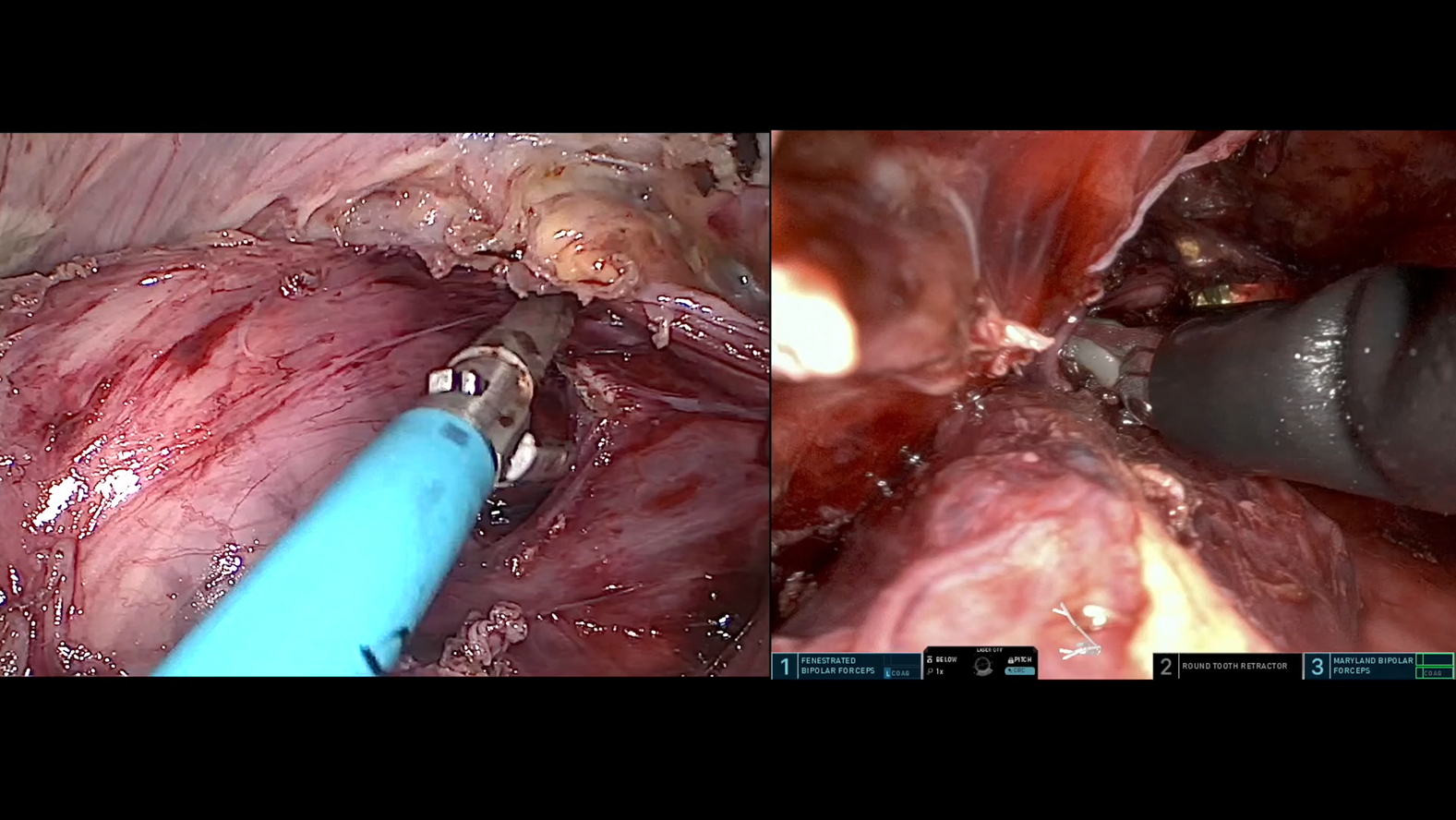

Supplement: Video 2 — Connecting the SP robotic mediastinal and laparoscopic abdominal phase. Video available at: https://www.jtcvs.org/article/S2666-2507(25)00323-2/fulltext. [file fx3.jpg]

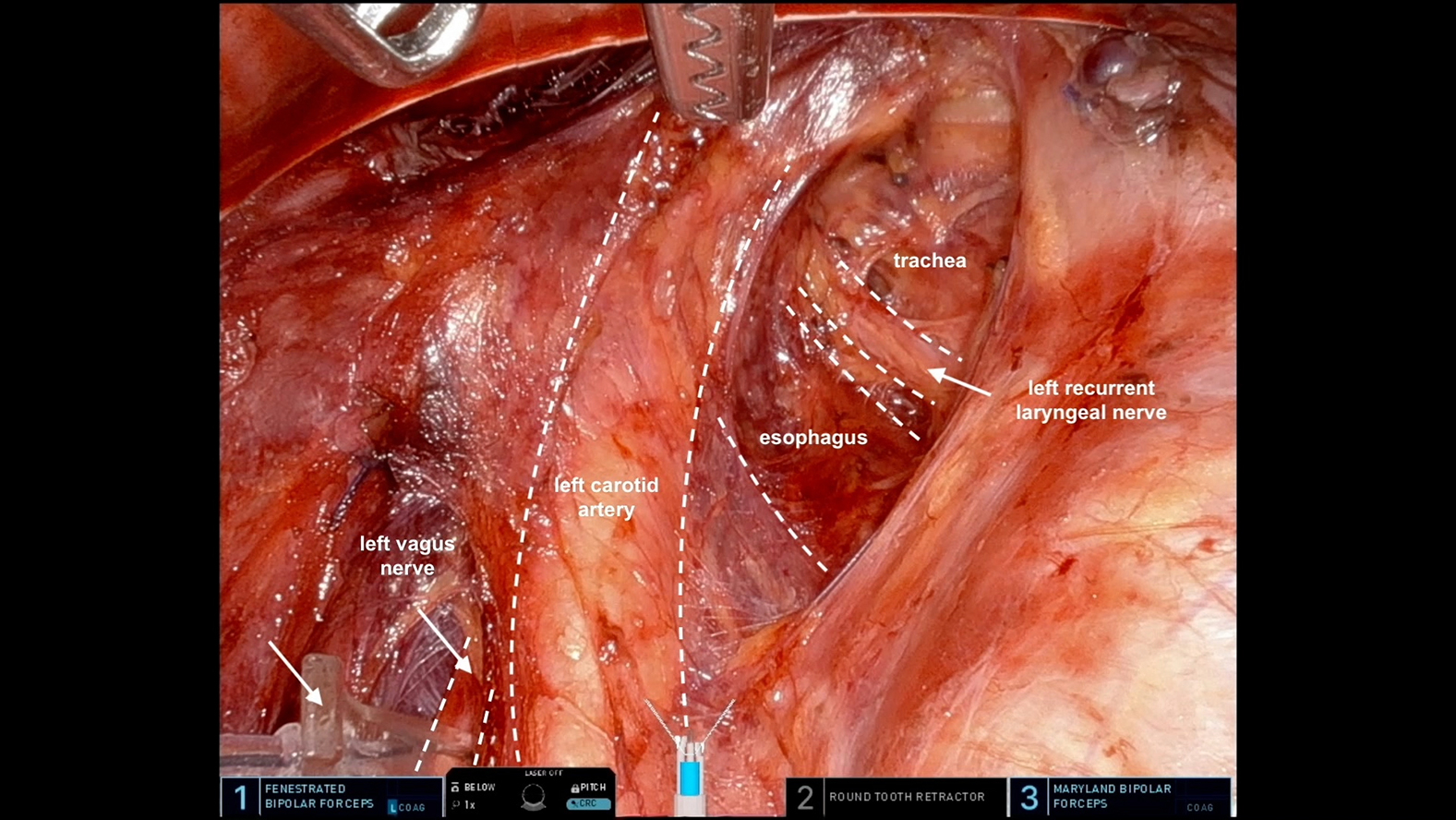

Supplement: Video 3 — SP RACE set-up, cervical access, and port placement. Video available at: https://www.jtcvs.org/article/S2666-2507(25)00323-2/fulltext. [file fx4.jpg]

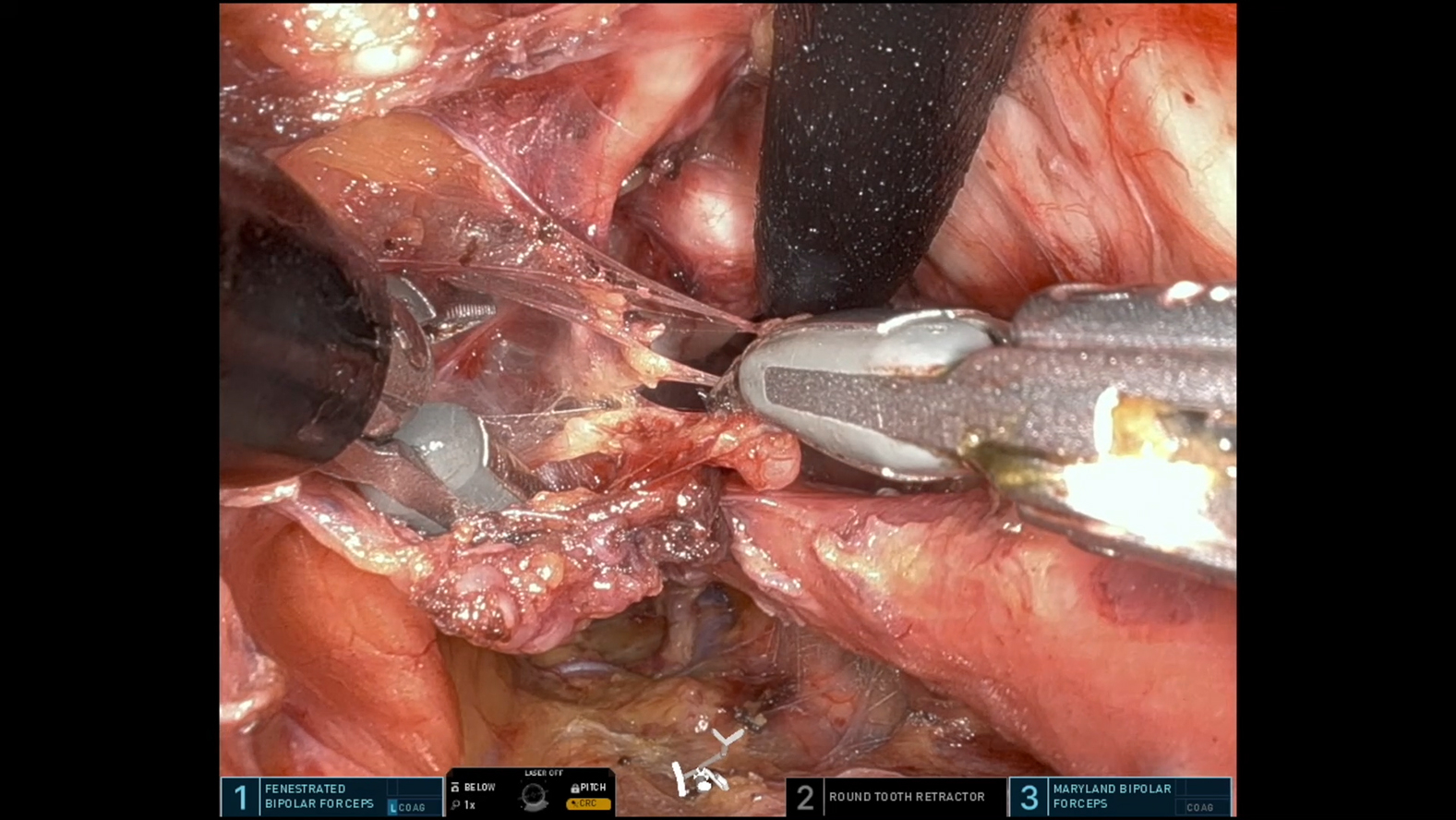

Supplement: Video 4 — SP robotic upper mediastinal left-sided esophageal dissection and left-sided recurrent laryngeal nerve lymphadenectomy. Video available at: https://www.jtcvs.org/article/S2666-2507(25)00323-2/fulltext. [file fx5.jpg]

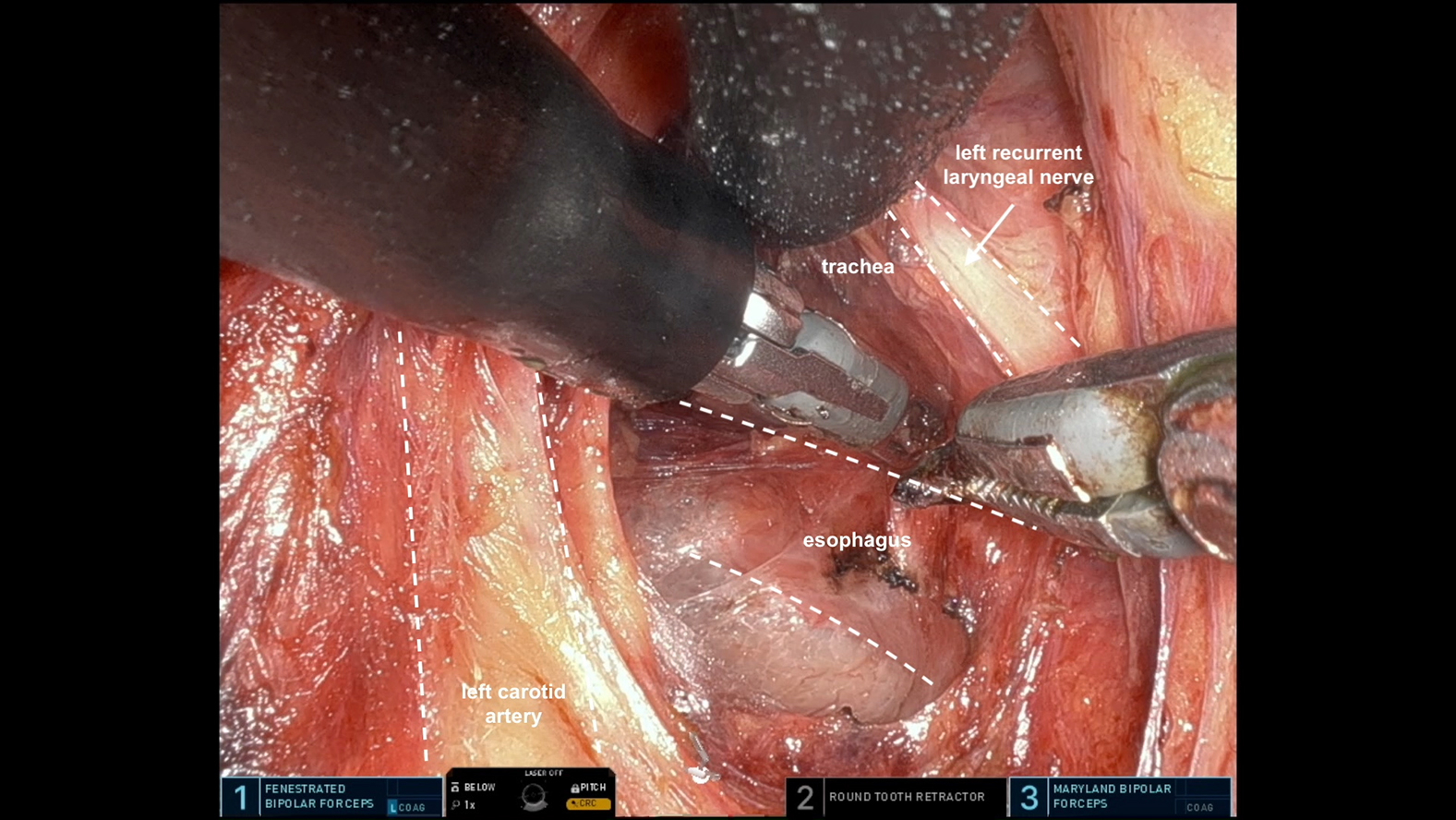

Supplement: Video 5 — SP robotic upper mediastinal dorsal esophageal dissection. Video available at: https://www.jtcvs.org/article/S2666-2507(25)00323-2/fulltext. [file fx6.jpg]

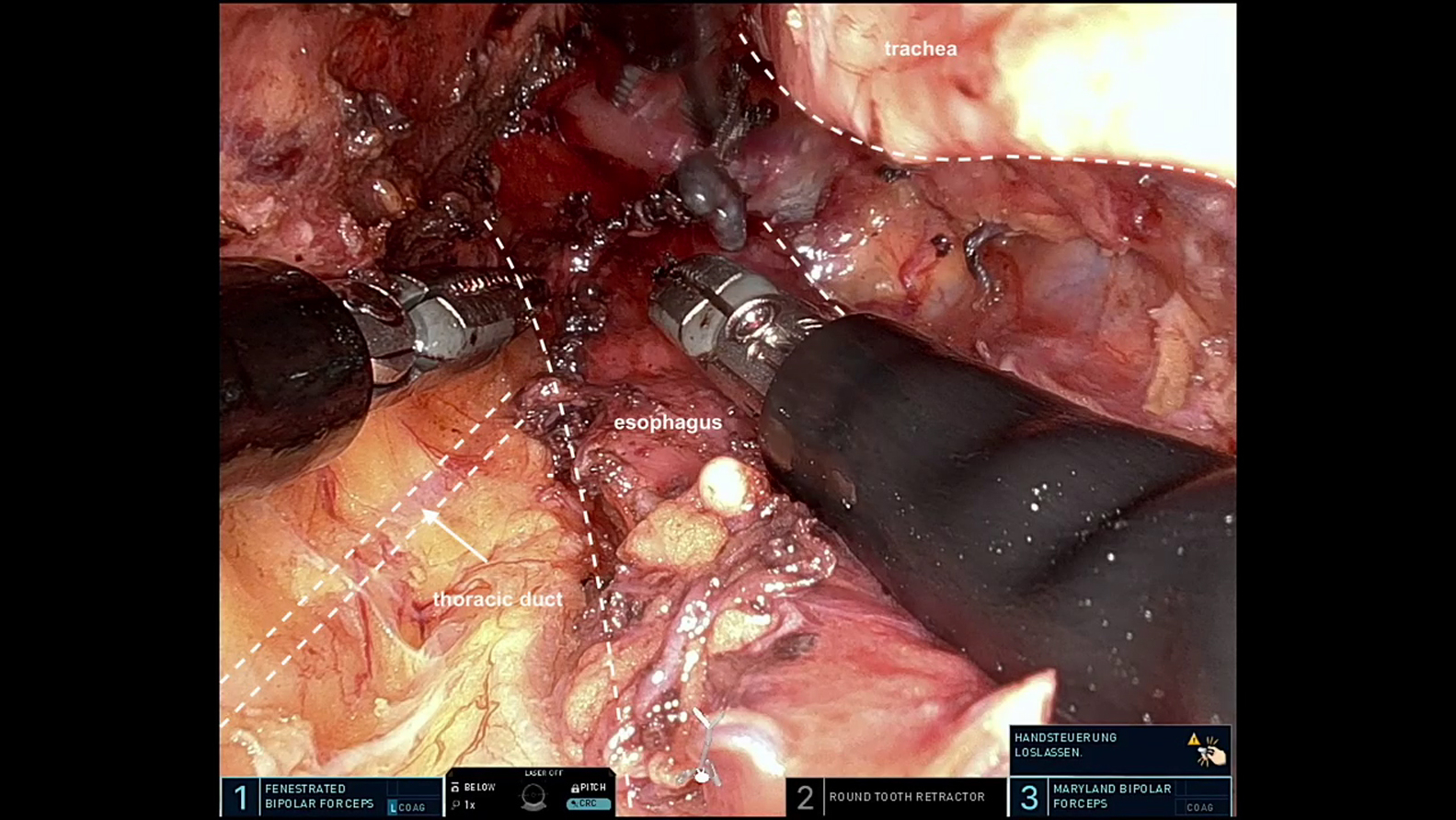

Supplement: Video 6 — Visualization of the thoracic duct with ICG. Video available at: https://www.jtcvs.org/article/S2666-2507(25)00323-2/fulltext. [file fx7.jpg]

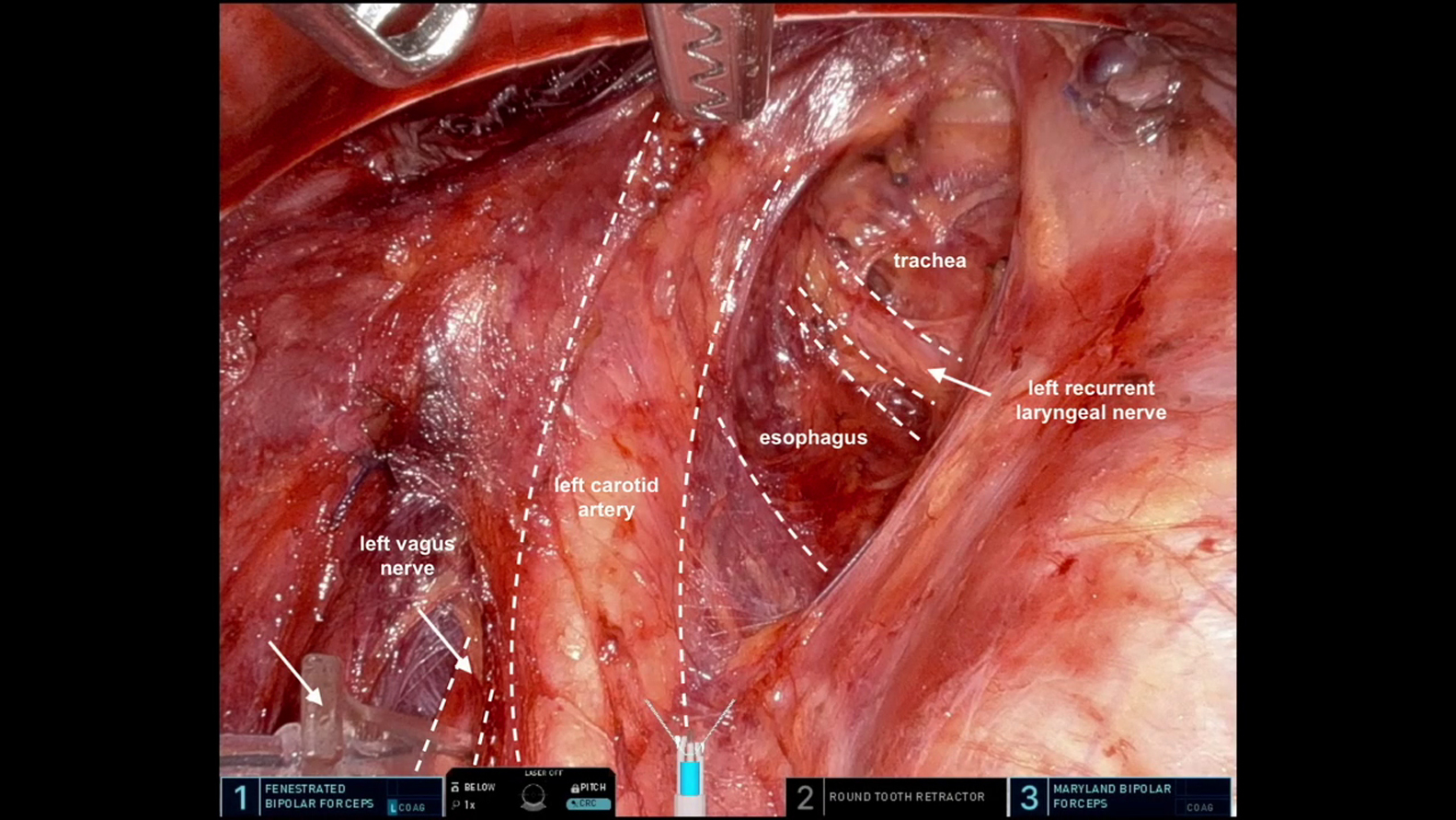

Supplement: Video 7 — SP robotic upper mediastinal ventral esophageal dissection. Video available at: https://www.jtcvs.org/article/S2666-2507(25)00323-2/fulltext. [file fx8.jpg]

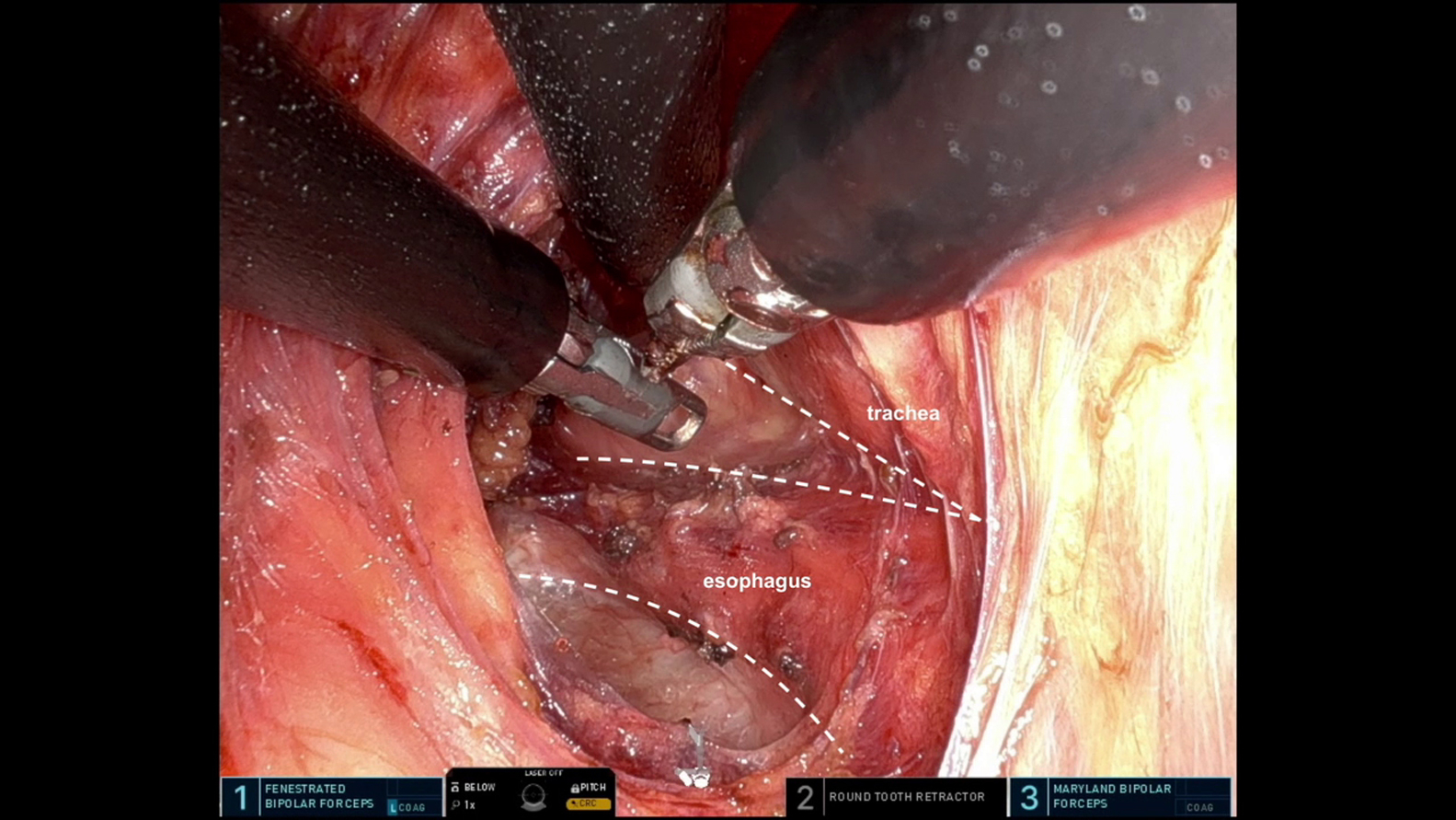

Supplement: Video 8 — SP robotic upper mediastinal right-sided esophageal dissection. Video available at: https://www.jtcvs.org/article/S2666-2507(25)00323-2/fulltext. [file fx9.jpg]

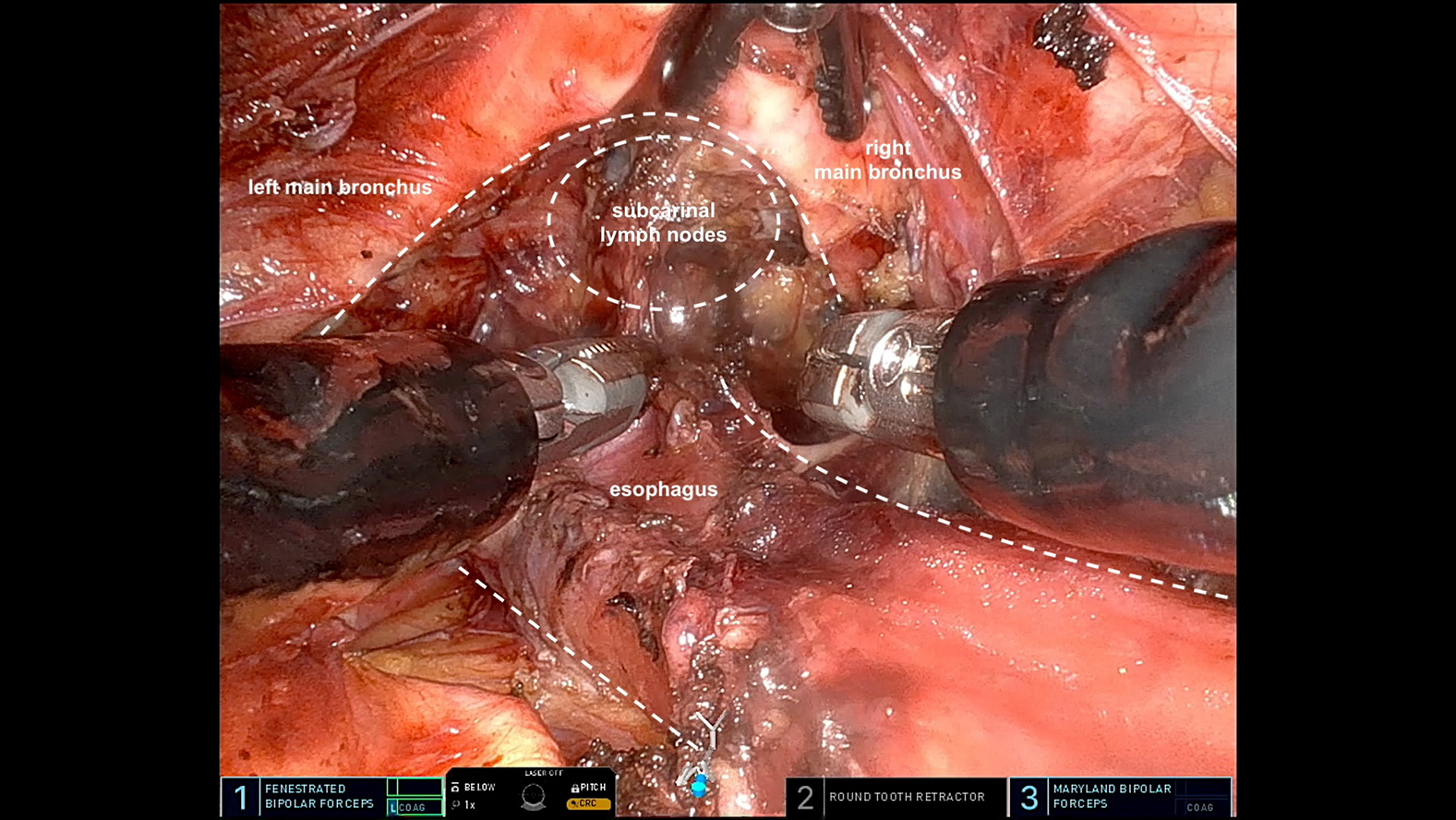

Supplement: Video 9 — SP robotic subcarinal lymphadenectomy. Video available at: https://www.jtcvs.org/article/S2666-2507(25)00323-2/fulltext. [file fx10.jpg]

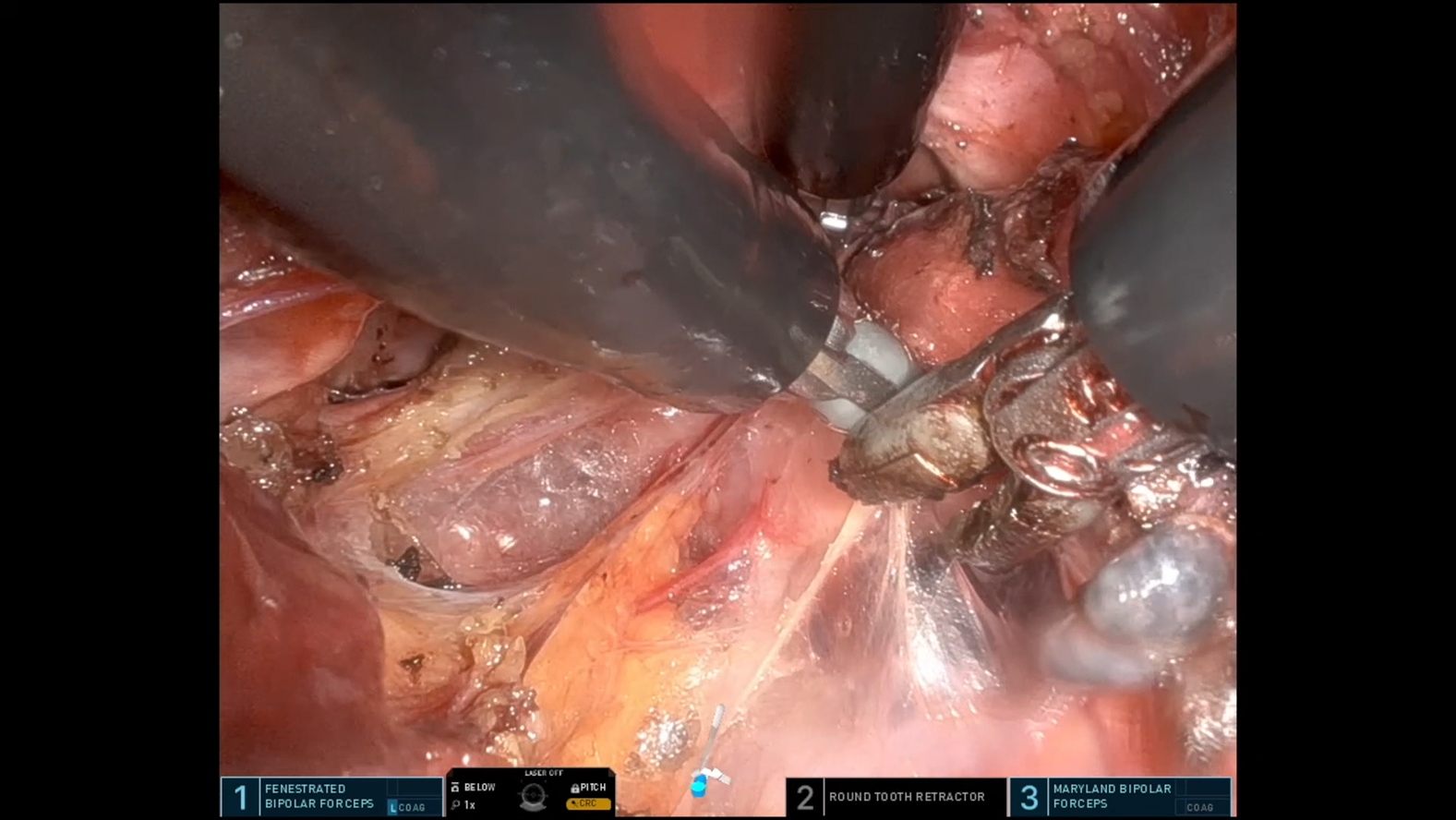

Supplement: Video 10 — SP robotic low mediastinal dissection of the esophagus. Video available at: https://www.jtcvs.org/article/S2666-2507(25)00323-2/fulltext. [file fx11.jpg]

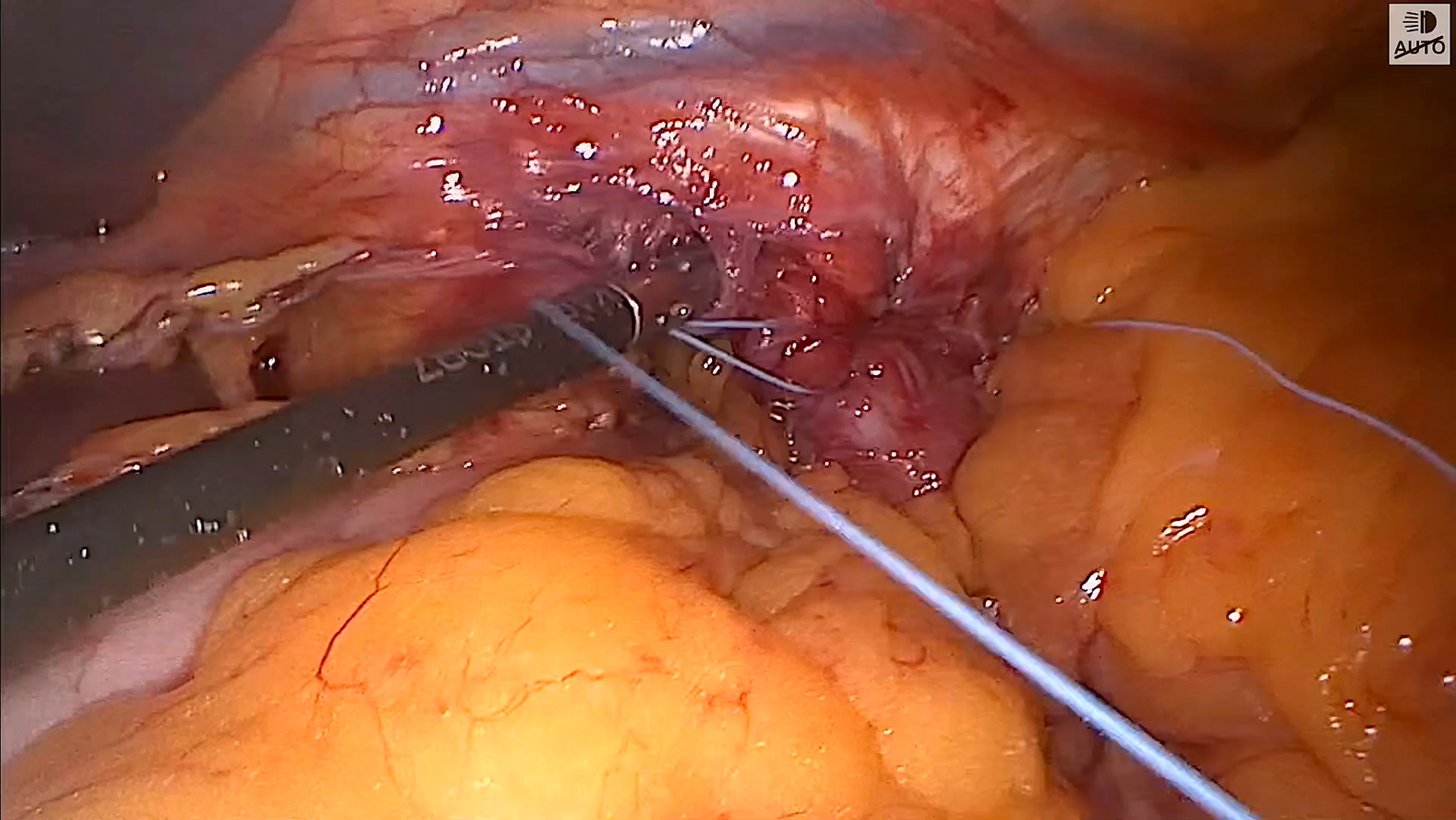

Supplement: Video 11 — Laparoscopic gastric conduit pull-up.Video available at: https://www.jtcvs.org/article/S2666-2507(25)00323-2/fulltext. [file fx12.jpg]
